# Reliability and validity of the German version of the Structured Interview of Personality Organization (STIPO)

**DOI:** 10.1186/1471-244X-13-210

**Published:** 2013-08-13

**Authors:** Stephan Doering, Markus Burgmer, Gereon Heuft, Dina Menke, Brigitta Bäumer, Margit Lübking, Marcus Feldmann, Susanne Hörz, Gudrun Schneider

**Affiliations:** 1 Department of Psychoanalysis and Psychotherapy, Medical University of Vienna, Währinger Gürtel 18-20, A-1090 Wien, Austria; 2 Department of Psychosomatics and Psychotherapy, University of Münster, Münster, Germany; 3 Private practice, Münster, Germany; 4 Private practice, Senden, Germany; 5 Alexianer Krankenhaus, Münster, Germany; 6 St. Vinzenz Hospital, Rhede, Germany; 7 Department of Psychology, Ludwig-Maximilians-Universität, Munich, Germany

**Keywords:** Personality functioning, Personality disorder, Diagnosis, Reliability, Validity

## Abstract

**Background:**

The assessment of personality organization and its observable behavioral manifestations, i.e. personality functioning, has a long tradition in psychodynamic psychiatry. Recently, the DSM-5 Levels of Personality Functioning Scale has moved it into the focus of psychiatric diagnostics. Based on Kernberg’s concept of personality organization the Structured Interview of Personality Organization (STIPO) was developed for diagnosing personality functioning. The STIPO covers seven dimensions: (1) identity, (2) object relations, (3) primitive defenses, (4) coping/rigidity, (5) aggression, (6) moral values, and (7) reality testing and perceptual distortions. The English version of the STIPO has previously revealed satisfying psychometric properties.

**Methods:**

Validity and reliability of the German version of the 100-item instrument have been evaluated in 122 psychiatric patients. All patients were diagnosed according to the Diagnostic and Statistical Manual for Mental Disorders (DSM-IV) and were assessed by means of the STIPO. Moreover, all patients completed eight questionnaires that served as criteria for external validity of the STIPO.

**Results:**

Interrater reliability varied between intraclass correlations of .89 and 1.0, Crohnbach’s α for the seven dimensions was .69 to .93. All a priori selected questionnaire scales correlated significantly with the corresponding STIPO dimensions. Patients with personality disorder (PD) revealed significantly higher STIPO scores (i.e. worse personality functioning) than patients without PD; patients cluster B PD showed significantly higher STIPO scores than patients with cluster C PD.

**Conclusions:**

Interrater reliability, Crohnbach’s α, concurrent validity, and differential validity of the STIPO are satisfying. The STIPO represents an appropriate instrument for the assessment of personality functioning in clinical and research settings.

## Background

The concept of personality organization or, in other terms, personality structure stands for intrapsychic formations that represent a basis of the personality and determine a person’s functioning in dealing with his or her own self and interpersonal relationships. Thus, personality functioning can be regarded as the observable manifestation of the underlying personality organization. The assessment of personality functioning goes back to Freud’s first structural model [1], that distinguished conscious, pre-conscious, and unconscious aspects of the mind. Based on Anna Freud’s work about the defense mechanisms [2] Hartmann [3,4] described ego functions as result of a healthy development and a basic condition for a mental equilibrium and psychosocial functioning. Kernberg [5,6] coined the term personality organization and initially distinguished three levels: Neurotic, borderline, and psychotic level of personality organization. While neurotic patients are characterized by an integrated identity, mature defense mechanisms (e.g., repression, rationalization, intellectualization), and good reality testing, borderline patients show impaired identity integration (“identity diffusion”) and primitive defense mechanisms (splitting, idealization, devaluation, denial, projective identification). (It has to be pointed out that the term borderline personality organization stands for a level of personality functioning and not for the nosological entity borderline personality disorder. However, borderline personality disorder usually occurs on a borderline level of personality organization.) On a psychotic level, in addition, reality testing is suspended. The basic assumption in Kernberg’s model is that the internal world of individuals on a borderline or psychotic level consists of split-off aspects of the self and others, which means that there are no integrated internal images of the self and significant others. This deficit leads to numerous problems in personality functioning in the realm of identity, interpersonal relations, coping with stress and aggression, as well as moral values. Kernberg presented a theoretical model that assigns the different personality disorders to different levels of personality organization [7], p. 14. In this model personality disorders like obsessive-compulsive or depressive-masochistic PD are on a neurotic level, histrionic, dependent, and narcissistic on a higher borderline level, and borderline, paranoid, schizoid, and antisocial on a lower borderline level of personality organization. Thus, DSM-IV cluster C personality disorders are located on a higher level of personality organization than cluster A and B personality disorders.

On the basis of Bowlby’s attachment theory [8] Fonagy [9,10] developed the model of mentalization that focuses on an individual’s ability to understand emotions, thoughts, and motives of other people and the mutual processes in interpersonal relationships. Mentalization has been operationalized as reflective functioning and can be assessed by means of Fonagy’s Reflective Functioning Scale [11]. It has been shown that reflective functioning is highly correlated with personality organization in terms of Kernberg [12]. Alternative and well-established measures for the assessment of personality functioning are, e.g., Wallerstein’s Scales of Psychological Capacities (SPC) [13] and the Operationalized Psychodynamic Diagnosis (OPD) [14].

Based on the above-mentioned concepts, recently an assessment of personality functioning was developed [15] that has been incorporated into the new DSM-5 classification [16]. The annex (Section 3) of the DSM-5 contains the Levels of Personality Functioning Scale (LPFS) for diagnosing personality disorders. The LPFS consists of two dimensions with two subdomains each: Self (identity and self-direction) and interpersonal (empathy and intimacy). This scale is provided for future research as an assessment tool for severity of personality disorders [16]. The International Classification of Diseases (ICD-11) will probably contain a similar measure [17].

Kernberg was the first to describe a clinical interview, the “Structural Interview” [6,18], that aimed at the assessment of personality organization in a clinical and qualitative way. With the aim to quantify Kernberg’s dimensions of personality organization, Clarkin, Kernberg, and colleagues at first created a questionnaire, The Inventory of Personality Organization (IPO) [19,20]. Thereafter, an interviewer-based instrument was developed by the same group, the Structured Interview of Personality Organization (STIPO) [21]. This structured 100-item interview contains seven domains that were derived from Kernberg’s conceptual work: (1) identity, (2) quality of object relations, (3) primitive defenses, (4) coping and rigidity, (5) aggression, (6) moral values, (7) reality testing and perceptual distortions.

The development of the STIPO was described in detail by Stern et al. [22]. They focused on the three scales identity, primitive defenses, and reality testing as the core domains of Kernberg’s model of personality organization. Interrater reliability was found to be satisfying (intraclass correlations .96 for identity, .97 for primitive defenses, and .72 for reality testing) as was internal consistency (Cronbach’s alpha .86 for identity, .85 for primitive defenses, and .69 for reality testing). Moreover, the STIPO dimension identity predicted positive and negative affect (assessed by the Schedule of Nonadaptive an Adaptive Personality, SNAP [23]), whereas the STIPO scale primitive defenses was correlated with aggression (assessed by the SNAP and the IPO) and cluster B personality traits according to DSM-IV [22]. Stern et al. [22] cautiously regarded their initial study on the STIPO as a preliminary empirical support of Kernberg’s model of personality organization. They recommended it to researches “interested in the empirical relation between psychoanalytically informed constructs, contemporary trait models of normal and disordered personality and their neurobehavioral underpinnings, and current personality disorder nosology” [22], p. 43. In addition, Stern and colleagues pointed out the necessity of a replication of their results in other studies with diverse samples.

Hörz [24] demonstrated the construct validity of the STIPO by generating a prototype of borderline personality organization that correlated significantly with corresponding clinical measures. In a treatment outcome study on borderline personality disorder [25] the STIPO demonstrated its sensitivity to change: patients with borderline personality disorder treated with Transference-Focused Psychotherapy (TFP) [7] showed a significantly higher improvement of personality organization than patients of the control group. Moreover, the STIPO was used as a severity measure of psychopathology, especially in personality disorders: It was shown that worse personality functioning as assessed by the STIPO goes along with more axis I and more axis II diagnoses [26].

Taken together, the STIPO is the only structured interview for the assessment of personality functioning. As such, it allows for the determination of specific psychopathology beyond symptoms. Since particularly psychodynamic treatments aim at the change of personality functioning, an instrument like the STIPO is needed to empirically demonstrate changes of this kind, like it was done in the study by Doering et al. [25]. From a conceptual point of view as pointed out by Stern et al. [22] the STIPO might be helpful to empirically test Kernberg’s model of personality organization. For these reasons it seemed worthwhile to translate the instrument into German language and to replicate and in part extend the findings of the above mentioned previous studies. In this study reliability and validity of the German version of the 100-item STIPO was evaluated in 122 psychiatric patients. SCID-I and -II interviews were used for diagnosing psychiatric disorders and for the determination of discriminant validity. Scales from eight well-validated questionnaires served as external criteria for the assessment of concurrent validity.

## Methods

### Study design

This study was approved by the ethics committee of the medical faculty of the Westfälische Wilhelms-University Münster, Germany. After receiving detailed information about the study all subjects gave written informed consent. Patients were diagnosed according to DSM-IV [27] by means of SCID-I and -II [28,29] and underwent a STIPO interview. An additional interview, the Operationalized Psychodynamic Diagnosis [14] was conducted, the results of which are not reported in detail here. In addition, all participants of the study completed eight questionnaires.

Two interviewers conducted the STIPO interviews, two different interviewers the OPD interviews. The interviewers were blind to the results of the other interview and questionnaire results. The STIPO interviewers received a one-day training in conducting and scoring the interview. After this and before the study interrater reliability was determined by calculating intraclass correlation (ICC) of the interviewers’ assessments of six video-taped STIPO interviews. These interviews represent expert rated training cases on different levels of personality organization that were unknown by the raters.

### Patients

125 psychiatric patients were recruited between September 2007 and November 2009 at a psychiatric hospital and a private practice in Münster (Germany). The sampling from two different settings was done for feasibility reasons only. Inpatients were recruited at six different wards of the Alexianer Hospital Münster, Germany, which is a large psychiatric hospital. Due to the exclusion criteria that had to be expected, psychiatric emergency units geriatric (dementia) units were not used for recruitment. All patients included into the study had been referred to the hospital voluntarily. Patients were asked to participate (a) in accordance with the time schedule of the interviewers and (b) with the aim to select a heterogeneous sample with regard to diagnoses. Outpatients were recruited at the private practice of one of the interviewers. During the recruitment period every new patient was asked to participate, if he/she fulfilled the inclusion criteria of the study. There were no incentives for participation in the study except for the fact that the results of the psychological tests were made available to the psychiatrists/psychologists in charge, if the patient gave his/her consent.

Inclusion criterion was the presence of a psychiatric disorder according to DSM-IV, exclusion criteria were: (1) cognitive deficits due to severe substance abuse, organic or psychotic disorder, mental retardation, or psychotropic medication, (2) acute suicidality, and (3) insufficient knowledge of the German language.

### Assessments

#### Structured Interview of Personality Organization (STIPO)

The STIPO [21] is a 100-item structured interview for the assessment of personality organization, i.e., personality functioning. For each item in the STIPO manual one or more questions are given that have to be read to the patient and – if necessary – explored in depth by the interviewer. Items are rated on a three point scale: 0=pathology absent, 1=some pathology, sub-threshold, and 2=significant to severe pathology. The interview covers seven domains with subdomains (see Table 1). Two scoring algorithms are provided: (1) For the arithmetic scoring the domains’ and subdomains’ mean values of the above mentioned three point ratings are calculated. (2) The second scoring algorithm employs a five-point scale from 1=good functioning to 5=severe impairment for the STIPO domains and subdomains. This rating is made by the interviewer on the basis of operationalizations of the five levels for each of the domains and subdomains. All STIPO domains and sub domains are scored separately, a higher scoring indicates more severe pathology.

**Table 1:** The domains and subdomains of the STIPO

**1. Identity**	A. Capacity to invest
	B. Sense of self
	a) Coherence and continuity
	b) Self valuation
	C. Sense of others
**2. Object relations**	A. Interpersonal relationships
	B. Intimate relationships and sexuality
	C. Internal working model of relationships
**3. Primitive defenses**	
**4. Coping / rigidity**	
**5. Aggression**	A. Self-directed aggression
	B. Other-directed aggression
**6. Moral values**	
**7. Reality testing and**	
**perceptual distortions**	

With the aim to depict Kernberg’s theoretical approach that distinguishes between neurotic and different levels of borderline personality organization, a final overall rating of personality organization is made on a six-point scale: (1) normal, (2) neurotic 1, (3) neurotic 2, (4) borderline 1, (5) borderline 2, and (6) borderline 3; the STIPO manual provides operationalizations for each of the six levels. Thus, a higher rating indicates greater pathology. Corresponding to Kernberg’s concept, the levels neurotic 1 and 2 stand for two levels of personality organization with an integrated identity and the use of mostly mature defense mechanisms. The levels Borderline 1 to 3 depict increasing degrees of identity diffusion and primitive defense mechanisms (i.e., splitting, idealization, devaluation, and projective identification), as well as self- and others-directed aggression, antisocial traits, and impaired reality testing. The level Borderline 3 comes close to what Kernberg conceptualized as psychotic personality organization; a psychotic level per se was not included, since it is not possible to conduct the STIPO in patients with a considerable impairment of reality testing.

The interview takes an average of 90 to 180 minutes, the rating is performed by the interviewer during the interview.

The original English version of the STIPO was translated into German, every item was discussed in detail by the authors and the translators, and the final German version was approved by the bilingual O.F. Kernberg, who is one of the authors of the English STIPO version. Both, English and German versions, are freely available on the internet (http://istfp.org/publications/diagnostic-instruments/).

#### Structured Clinical Interview for DSM-IV (SCID)

SCID-I and -II [28,29] are the official diagnostic tools for the Diagnostic and Statistical Manual of Psychiatric Disorders [27]. SCID-I assesses symptom disorders, SCID-II personality disorders. The instrument is established as gold standard for the reliable assessment of psychiatric disorders. Interrater reliability for SCID-I was above .70 for mood, anxiety, schizophrenic disorders, and alcohol abuse; it was somewhat lower for a few other disorders [30], for SCID-II it was reported between .48 and .98 for the categorical diagnoses (Cohen’s κ) and .90 to .98 for the dimensional judgements (intraclass correlation coefficient) [31]. Crohnbach’s α was found between .71 and .94 for the SCID-II personality disorder scales [31]. The duration of the SCID-I is 30 to 90 minutes, the duration of the SCID-II is 30 to 60 minutes.

#### Operationalized Psychodynamic Diagnosis (OPD-2)

The OPD-2 [14] represents a multidimensional interview-based diagnosis of psychiatric disorders. In addition to the symptomatic diagnosis according to ICD-10 [32] or DSM-IV [27] four axes are provided: (1) experience of illness and prerequisites for treatment, (2) interpersonal relations, (3) conflict, and (4) structure. Axis 4 is designed for the evaluation of psychic structure and was used for the determination of convergent validity of the STIPO in this study. It contains eight domains: (1) cognitive ability: self-perception, (2) cognitive ability: object perception, (3) capacity for regulation: self-regulation, (4) capacity for regulation: regulation of object relationship, (5) emotional ability: internal communication, (6) emotional ability: communication with the external world, (7) attachment capacity: internal objects, and (8) attachment capacity: external objects. Based on detailed operationalizations the eight dimensions are rated on a seven-point scale of structural integration (1=high, 1.5, 2=moderate, 2.5, 3=low, 3.5, 4=disintegrated). Finally, a seven-point general assessment of psychic structure is provided. Interrater reliability was found to vary between .61 and .82 (Cohen’s κ) for the dimensions and .83 for the total score [33]. Internal consistency (Crohnbach’s α) for the OPD-2 structure axis was reported to be .87 [34].

#### Borderline Personality Inventory (BPI)

The BPI (German: Borderline Persönlichkeitsinventar) [35] was developed for the assessment of personality organization (i.e., personality functioning) according to Kernberg’s theory [6]. The questionnaire contains 53 dichotomous items which cover the dimensions (1) identity diffusion, (2) primitive defense mechanisms and object relations, (3) reality testing, and (4) fear of closeness. The internal consistency of the BPI scales varies between .68 and .91, re-test reliability was sufficient (> .80) for all scales except the reality testing scale, and the convergent validity with other related instruments was shown to be satisfactory [35,36].

Although the scales are named similar to the STIPO scales, there are some important differences: The BPI scale identity diffusion covers depersonalization, which is part of the STIPO dimension reality testing; the BPI scale primitive defenses and object relations embraces the content of two corresponding STIPO scales; the BPI scale reality testing aims exclusively at psychotic symptoms whereas the corresponding STIPO dimension also covers perceptual distortions; the BPI scale fear of closeness focuses only one aspect of the broader defined STIPO dimension object relations.

#### Stress-Process Questionnaire (SPQ)

The Stress-Process Questionnaire (SPQ; German: Stressverarbeitungsfragebogen, SVF-120) [37] is a 120-item instrument that covers 20 subscales with six items each. Ten positive coping subscales are combined to three domains: (1) devaluation/ defense, (2) distraction, and (3) control of stressor. Six subscales are subsumed under a negative coping domain, and four scales describe different domains: (1) need for social support, (2) avoidance, (3) aggression, and (4) taking medication. Internal consistency was determined between .66 and .92 for the SPQ scales, re-test reliability ranged between .69 and .86, and numerous studies demonstrated construct validity of the instrument [37].

#### State-Trait-Anger-Expression-Inventory (STAXI)

The STAXI (German: State-Trait-Ärgerausdrucks-Inventar) [38,39] represents a well-established 44-item self-rating instrument for the assessment of state and trait anger as well as three different types of anger expression: (1) anger out (describes the amount of the overt and direct expression of anger), (2) anger in (covers avoidance of anger expression and the suppression of anger), and (3) anger control (evaluates the ability to control feelings and expression of anger). The internal consistency of the questionnaire was shown to be good (.75 to .95 for the three scales), a satisfactory convergent and discriminant validity was shown in a number of studies [38,39].

#### Dissociative Experiences Scale (DES)

The questionnaire (German: Fragebogen zu dissoziativen Symptomen, FDS-20) [40] represents a modified German version of the Dissociative Experiences Scale (DES) [41]. The 44-item questionnaire contains four subscales: (1) amnesia, (2) absorption, (3) derealisation, and (4) conversion. In this study the 20-item short version (FDS-20) was employed. Both German versions of the instrument yielded good reliability and validity. The internal consistency of the FDS-20 is .93, re-test reliability was found to be .80 to .82. [40].

#### Coping with Conflict Questionnaire

The questionnaire (German: Fragebogen zu Konfliktbewältigungsstrategien, FKBS) [42] was developed in the style of the Defense Mechanism Inventory (DMI) [43]. It assesses defense and coping styles by means of ten short narrations of conflictual social situations. Subjects have to judge 10 possible emotional and behavioral reactions of one of the acting persons on a four point scale. The reporting reveals five dimensions: (1) reversal, (2) turning against self, (3) turning against object, (4) intellectualisation/ rationalisation, and (5) projection. The German version of the questionnaire shows an internal consistency of .78 to .90 for the scales and a re-test reliability of .71 to .84 [42]. Moreover, sufficient convergent validity was demonstrated [42].

#### Frankfurt Self-Concept Scales

The questionnaire (German: Frankfurter Selbstkonzeptskalen, FSKN) [44] was developed to assess an individual’s attitudes, cognitions, emotions, and behavior towards him- or herself. It is assumed that a stable personality goes along with a positive self-concept. The 78 items of the self-rating instrument are answered on a six-point scale. The questionnaire yields ten subscales representing different self-concepts: (1) general fitness, (2) general ability to solve problems, (3) confidence concerning conduct and decisions, (4) general self-esteem, (5) sensitivity and mood, (6) firmness against others, (7) contact and ability to communicate, (8) esteem by others, (9) irritability by others, and (10) feelings and relations to others. The test shows satisfactory internal consistency, re-test reliability after four to five month was shown to be .82 [44].

#### Experiences in Close Relationships (ECR)

The ECR (German: Bochumer Bindungsfragebogen, BoBi) [45,46] aims at the assessment of attachment in intimate relationships. 36 Items are rated on a 7-point scale. Attachment is evaluated on the two dimensions anxiety and avoidance with low scores in healthy persons. Reliability and validity of the German version of the questionnaire have been reported to be satisfying, internal consistency was .85 (avoidance) and .91 (anxiety) [46].

#### Assessment of DSM-IV Personality Disorders (ADP-IV)

The ADP-IV [47] was originally published in Dutch and translated into German. The 94 items of the questionnaire cover the same number of diagnostic items as the DSM-IV [27] personality disorders. Every item is rated on a seven-point scale. In addition, once an item is rated 5 or above, a three-point distress rating has to be completed. The report contains a categorical as well as a dimensional assessment of DSM-IV personality disorder diagnoses. The German version of the instrument yielded good reliability. The categorical diagnoses are not sufficiently valid for a clinical diagnosis, but dimensional ratings show a satisfactory sensitivity, thus, the instrument can be recommended for screening purposes [48,49]. The instrument was employed in addition to the SCID-II, because of its higher dimensional sensitivity particularly in the realm below the threshold for the categorical DSM-IV diagnosis of antisocial personality disorder.

### Hypotheses

For the determination of the construct validity correlations of the STIPO domains with specific scales of the questionnaires were hypothesized a priori:

a) STIPO “identity” correlates positively with BPI “identity diffusion”.

b) STIPO “object relations” correlates positively with BPI “fear of closeness” as well as ECR “avoidance” and “anxiety”. It correlates negatively with FSKN “contact and ability to communicate” and “feelings and relations to others”.

c) STIPO “primitive defenses” correlates positively with BPI “primitive defense mechanisms and object relations” and FKBS “Projection”.

d) STIPO “coping/rigidity” correlates negatively with SPQ “Positive coping” and positively with SPQ “Negative coping” as well as negatively with FSKN “general ability to solve problems”.

e) STIPO “self-directed aggression” correlates positively with STAXI “anger in”, and FKBS “turning against self”; STIPO “other-directed aggression” correlates positively with STAXI “anger out” and FKBS “turning against others”.

f) STIPO “moral values” correlates positively with ADP-IV “antisocial personality disorder” (dimensional score).

g) STIPO “reality testing and perceptual distortions” correlates positively with FDS-20 total mean score and BPI “reality testing”.

h) STIPO overall rating correlates positively with the OPD total score of structural integration.

Moreover, it was hypothesized that the STIPO scores differ significantly between patients with cluster A, B, and C personality disorders, even after controlling for severity by means of the GAF score (discriminant validity). This assumption is in line with Kernberg’s assumption of the correlation between personality disorders and personality organization:

i) Patients with cluster A and cluster B personality disorder, respectively, reveal a worse personality organization in terms of a higher total STIPO score than those with cluster C personality. These differences remain significant after controlling for general severity of psychopathology (GAF score).

It was assumed that the different STIPO domains intercorrelate to some degree, since according to Kernberg’s model they are all based in a unifying, underlying construct, i.e. personality organization.

### Statistics

Cronbachs α was determined for the STIPO dimensions. Interrater reliability was evaluated by means of intraclass correlations (ICC). To test the hypotheses of construct validity, Spearman correlations were calculated. T-tests were used for group comparisons (discriminant validity). IBM SPSS Statistics 20.0 (IBM Corporation, Armonk, New York, USA) was employed.

## Results

### Sample characteristics

Three patients had to be excluded from the study, because they did not complete the STIPO interview, thus, 122 patients were included into the analyses. Ninety-one patients (74.6%) were psychiatric inpatients, 31 (25.4%) outpatients of a psychotherapists’ private practice. Demographic data and diagnoses according to DSM-IV [27] are given in Table 2. On axis I 1.6% received no diagnosis, 44.3% had one diagnosis, 54.1% more than one diagnosis. On axis II 36.9% had no diagnosis, 29.5% one, and 33.6% more than one diagnosis.

**Table 2:** Sample characteristics (n=122)

	**Mean (s.d.)**
**Age (years)**	40.95 (14.61)
	range: 18-70
	**n (%)**
**Gender**	
Female	85 (69.7)
Male	37 (30.3)
**Education**	
No compulsory school	1 (0.8)
Compulsory school	8 (6.6)
Apprenticeship/ vocational school	36 (29.5)
High school	28 (23.0)
A-level	33 (27.0)
Academic	9 (7.4)
Other	4 (3.3)
Missing	3 (2.5)
**Employment**	
In occupational training	13 (10.7)
Unemployed	30 (24.6)
Part-time	19 (15.6)
Full-time	32 (26.2)
Homemaker	7 (5.7)
Retired (due to disorder)	17 (13.9)
Missing	4 (3.3)
**Family status**	
Single	36 (29.5)
Unmarried with partner	16 (13.1)
Married	44 (36.1)
Divorced/ separated	18 (14.8)
Widowed	3 (2.5)
Missing values	5 (4.1)
Diagnoses on DSM-IV axis I*	**n**
Substance abuse disorders	34
Mood disorders	105
Brief psychotic disorder	1
Anxiety disorders	37
Posttraumatic stress disorder	16
Obsessive-compulsive disorder	6
Somatoform disorders	12
Eating disorders	11
Other	10
Diagnoses on DSM-IV axis II (personality disorders)*	**n**
Paranoid	14
Schizoid	4
Schizotypal	1
Obsessive-compulsive	20
Histrionic	4
Dependent	14
Antisocial	13
Narcissistic	15
Avoidant	23
Borderline	36
Depressive	8
Passive-aggressive	3
**STIPO level of personality organization**	**n (%)**
1 – normal	3 (2.5)
2 – neurotic 1	20 (16.4)
3 – neurotic 2	38 (31.1)
4 – borderline 1	38 (31.1)
5 – borderline 2	22 (18.0)
6 – borderline 3	1 (0.8)

*More than one diagnosis per patient included.

### Interrater reliability

Two interviewers delivered the STIPO interviews and performed the ratings after having received a comprehensive one day rater training. Interrater reliability was determined by calculating intraclass correlation (ICC) of the interviewers’ assessments of six video-taped STIPO interviews.

ICC of the five-point ratings varied between .89 and 1.0 for the seven dimensions and was .96 for the global rating of personality organization. The ICC for the arithmetic ratings varied between .88 and .98 for the seven dimensions.

### Internal consistency

Crohnbach’s α was calculated for the seven STIPO scales: identity (30 items) α=.93, quality of object relations (22 items) α=.88, primitive defenses (11 items) α=.88, coping and rigidity (10 items) α=.80, aggression (12 items) α=.83, moral values (8 items) α=.84, reality testing and perceptual distortions (7 items) α=.69. Cronbach’s α for the total score calculated from all 100 items was α=.97.

### Correlations of the different STIPO rating algorithms

The correlations between two different scoring algorithms (arithmetic and five-point scale) were .85 for the identity dimension, .75 for object relations, .91 for primitive defenses, .80 for coping/rigidity, .87 for aggression, .86 for moral values, and .82 for reality testing.

### Intercorrelation of STIPO dimensions

All STIPO dimensions correlate significantly among each other. The correlations vary between .48 and .79 (see Table 3).

**Table 3:** Intercorrelations among STIPO domains (5-point ratings) and corresponding questionnaire scales

	**STIPO domains**							
	**Identity**	**Object**	**Primitive**	**Coping/**	**Self-directed**	**Other-directed**	**Moral**	**Reality**
		**relations**	**defenses**	**rigidity**	**Aggression**	**Aggression**	**values**	**testing**
STIPO – Identity	-							
STIPO – Object relations	.64**	-						
STIPO – Primitve defenses	.79**	.58**	-					
STIPO – Coping/rigidity	.70**	.54**	.72**	-				
STIPO – Self-directed Aggression	.51**	.41**	.55**	.47**	-			
STIPO – Other-directed Aggression	.48**	.48**	.57**	.50**	.45**	-		
STIPO – Moral values	.49**	.50**	.53**	.52**	.47**	.51**	-	
STIPO – Reality testing	.62*	.59**	.69**	.64**	.52**	.61**	.57**	-
BPI – Primitive defenses	.45**	.35**	.49**	.43**	.36**	.38**	.27**	.39**
BPI – Identity diffusion	.44**	.35**	.52**	.43**	.49**	.47**	.25**	.46**
BPI – Fear of closeness	.48**	.39**	.54**	.46**	.46**	.40**	.27**	.41**
BPI – Reality testing	.36**	.23*	.40**	.20*	.23**	.25**	.14	.34**
FSKN – Contact, communication	-.30**	-.21*	-.19*	-.27**	-.24*	-.18	-.08	-.28**
FSKN – Feelings, relations	-.37**	-.35**	-.27**	-.34**	-.27**	-.27**	-.19*	-.28**
FSKN – Problem solving	-.22*	-.13	-.22*	-.19*	-.21*	-.20*	-.21*	-.12
ECR - Avoidance	.32**	.33**	.34**	.17	.32**	.07	.15	.30**
ECR - Anxiety	.27**	.25*	.28*	.24*	.27*	.44**	.28*	.21
FKBS - Projection	.29**	.23*	.37**	.34**	.32*	.37**	.22*	.27**
FKBS – Turning against self	.27**	.22*	.32**	.38**	.33**	.29**	.20*	.33**
FKBS – Turning against object	.29**	.23*	.37**	.30**	.36**	.40**	.29**	.32**
SPQ – Positive Coping	-.33**	-.21*	-.32**	-.33**	-.48**	-.25**	-.34**	.41**
SPQ – Negative Coping	.27**	.30**	.36**	.40**	.37**	.33**	.26**	.17
STAXI – Anger in	.23*	.22*	.22*	.28**	.23*	.23*	.15	.32**
STAXI – Anger out	.28**	.20*	.35**	.30**	.29**	.49**	.32**	.49**
ADP-IV – Antisocial PD	.51**	.51**	.55**	.40**	.43**	.48**	.54**	.47**
DES – Total score	.38**	.34**	.47**	.34**	.40**	.37**	.20*	.47**

Hypothesized correlations are printed bold (* p<.05, ** p<.01).

### Correlations of STIPO and questionnaire scales

Table 3 shows the correlations of the STIPO domains and the a priori selected questionnaire scales. All correlations are significant in the predicted direction. The correlations vary between .19 and .60. Most of them can be regarded as medium size (0.3 to 0.5).

Correlations occurred not only between the corresponding scales according to the a priori hypotheses, but also between theoretically less closely related scales. In general, the correlations were highest between the STIPO scales and the BPI scales as well as the ADP-IV antisocial PD scale. Here the results are given with regard to the a priori hypotheses:

a) As predicted, STIPO “identity” correlates positively with BPI “identity diffusion”, but the correlations with two other BPI scales (“primitive defenses” and “fear of closeness”) and the ADP-IV “antisocial PD” scale are equally high.

b) STIPO “object relations” correlates positively with the predicted BPI “fear of closeness”, but the correlations with the other predicted scales are lower. Again, the correlation with the ADP-IV “antisocial PD” scale is the highest of all.

c) STIPO “primitive defenses” correlates positively with the predicted BPI “primitive defense mechanisms” and “object relations”, the correlation with the predicted FKBS “projection” was also significant, but considerably lower. Again, the highest correlations occurred with two BPI scales (“identity diffusion” and “fear of closeness”) as well as with the ADP-IV “antisocial PD” scale and the DES total score.

d) STIPO “coping/rigidity” shows a high correlation with SPQ “negative coping”. The negative correlation with SPQ “positive coping” is somewhat lower, but also in the predicted direction. Similar to the previously mentioned dimensions the highest correlations occurred with the BPI dimensions (except “reality testing”) and the ADP-IV “antisocial PD” scale.

e) STIPO “self-directed aggression” shows the highest correlations with BPI “identity diffusion” and “fear of closeness”, SPQ “positive coping” (negatively correlated), ADP-IV “antisocial PD” scale, and DES total score. The expected correlations with FKBS “turning against self” and STAXI “anger in” where significant, but somewhat lower. STIPO “other-directed aggression” revealed the predicted correlations with STAXI “anger out” and FKBS “turning against others”, but also correlated highly with BPI “identity diffusion” and “fear of closeness”, ECR “anxiety”, and ADP-IV “antisocial PD” scale.

f) As expected, STIPO “moral values” showed the highest correlation with the ADP-IV “antisocial PD” scale.

g) STIPO “reality testing” correlated expectedly high with the FDS-20 total score, but somewhat lower with BPI “reality testing”. Moreover, high correlations occurred with BPI “identity diffusion” and “fear of closeness”, SPQ “positive coping”, STAXI “anger out”, and ADP-IV “antisocial PD” scale.

### Correlation of STIPO and OPD total score

The STIPO level of personality organization correlated significantly with the overall rating of the OPD axis 4 total score (r=.68, p<.001).

### Correlations of STIPO and diagnosis of personality disorder

Table 4 shows the differences between patients with and without personality disorder on the STIPO domains. Patients with personality disorder revealed significantly lower level of personality organization in all domains compared to patients without personality disorder. The between group effect size for the total score was d=1.62.

**Table 4:** STIPO ratings (5-point ratings) of personality organization in patients with and without personality disorder (T-test)

**STIPO domain**	**Personality disorder**				**Statistics**		
	**No (n=45)**		**Yes (n=77)**				
	***Mean***	***s.d.***	***Mean***	***s.d.***	***T***	***df***	***p***
Identity	1.93	.69	3.06	.76	−8.160	120	<.001
Object relations	2.13	.66	3.06	.83	−6.401	120	<.001
Primitve defenses	1.80	.92	3.22	.77	−9.135	120	<.001
Coping/rigidity	2.29	.59	3.32	.73	−8.071	120	<.001
Aggression	1.78	.67	2.70	.92	−5.886	120	<.001
Moral values	1.47	.55	2.25	.80	−5.807	120	<.001
Reality testing and perceptual distortions	1.20	.41	2.09	.57	−9.258	120	<.001
Total score	2.62	.86	3.99	.84	−8.614	120	<.001

In addition, patients with cluster B personality disorders only (borderline, histrionic, narcissistic, antisocial) were compared to patients with cluster C personality disorders only (avoidant, dependent, obsessive-compulsive) (see Table 5). Since only one patient had a sole cluster A personality disorder, this group was not included into the analysis. Cluster B patients yielded a significantly lower level of personality organization in all but two domains (d=1.29 between group effect size for the total score).

**Table 5:** STIPO ratings (5-point ratings) of personality organization in patients with only cluster B and only cluster C personality disorder (T-test)

**STIPO domain**	**Personality disorders**				**Statistics**		
	**Cluster B (n=29)**		**Cluster C (n=19)**				
	***Mean***	***s.d.***	***Mean***	***s.d.***	***T***	***df***	***p***
Identity	3.34	.81	2.66	.63	3.121	46	.003
Object relations	3.26	.83	2.53	.70	3.177	46	.003
Primitve defenses	3.55	.69	2.58	.69	4.787	46	<.001
Coping/rigidity	3.41	.73	3.11	.81	1.369	46	.178
Aggression	3.10	.86	2.19	.77	3.889	46	<.001
Moral values	2.59	.78	1.84	.60	3.523	46	.001
Reality testing and perceptual distortions	2.14	.69	1.84	.50	1.603	46	.116
Total score	4.34	.77	3.37	.76	4.320	46	<.001

To make sure that the STIPO assesses personality organization and not just general psychosocial functioning, all of these analyses were controlled for psychosocial functioning in terms of the Global Assessment of Functioning Score (GAF) of the DSM-IV [27] by means of analyses of covariance (ANCOVA). All comparisons remained significant for the comparisons between patients with and without personality disorder. In cluster B vs. cluster C personality disorders the results remained significant for the total score and all but three STIPO dimensions; for the dimensions object relations, coping/rigidity, and reality testing the level of significance was reduced to a trend niveau (p>.01).

## Discussion

The Structured Interview of Personality Organization was evaluated with regard to interrater reliability, internal consistency of the seven scales, as well as concurrent and discriminant validity.

Interrater reliability was high, the intraclass correlations (ICC) of .89 to 1.0 for the seven dimensions and .96 for the global rating are in line with those reported by Stern et al. [22] for the STIPO as well as with other structured interviews like the SCID-II (.90 to .98) [31]. As to be expected, the interrater reliability of this structured interview exceeds the numbers reported for more unstructured, clinically oriented interviews like the Scales of Psychological Capacities (SPC) [13] or the Operationalized Psychodynamic Diagnosis (OPD-2) [14]. For the SPC ICC of .54 to .89 (mean ICC=.82) were reported [50], for the OPD-2 structure axis, the ICC varied between .61 and .82 for the subdimensions and .83 for the total score [33]. This difference can easily be explained by the fact that structured interviews give more detailed and strict advice for the rating of each single item, whereas unstructured interviews grant the freedom to judge in a more clinical fashion, which might lead to a better clinical impression of the patient at the expense of reliability in terms of agreement between different raters.

The internal consistency of the seven STIPO dimensions was found between .80 and .93 with the exception of the dimension reality testing (.69), Crohnbach’s α for the total score was .97. This confirms the numbers reported by Stern et al. [22], who also found Crohnbach’s α above .80 for identity and primitive defenses, but lower in reality testing (.69). The lower internal consistency of the dimension reality testing can be explained by the fact that this scale contains different constructs like paranoid thinking, dissociation, and depersonalization that do not necessarily correlate highly in all patients. Maffei et al. [31] found Crohnbach’s α between .71 and .94 for the SCID-II personality disorder scales. These results show that the constructs of dimensions of personality functioning are as coherent as the constructs of distinct personality disorders in the DSM-IV; both are on a satisfactory level.

High correlations among the STIPO dimensions occurred (.48 to .79) which means that the seven dimensions are not independent from each other. This is not astonishing since Kernberg conceptualized the dimensions of personality organization as different manifestations of an underlying core pathology, namely identity diffusion as a result of disturbed development during early life due to genetic disposition and mainly adverse early relationships [5,6]. From a theoretical point of view it could be argued that one dimension would be enough for the determination of personality organization or functioning. This argument supports the development of a short version of the STIPO, which is currently being prepared by the authors of the instrument. From a clinical point of view one would be reluctant to relinquish the important detailed clinical information from each of the STIPO dimensions. As a consequence it will be recommendable to maintain both, a short and a long version; a short version for screening purposes and general scientific use and a long version for treatment planning in the clinical field and specific research questions.

As far as concurrent validity is concerned, the STIPO correlates significantly with all a priori selected corresponding questionnaire scales, but the STIPO dimensions also correlated significantly with almost all of the other questionnaire scales. At first, this result suggests, that a general factor underlies the different measures, which might be a general severity of psychopathology. However, a closer look reveals a number of relevant details of the correlational patterns. Throughout all STIPO dimensions except moral values and in part object relations and reality testing, the highest correlations occur with the BPI dimensions (except BPI reality testing). This can be attributed to the fact that the BPI is the only questionnaire employed in this study that is explicitly rooted in Kernberg’s theory, while other instruments like the Frankfurt Self-Concept Scales, the State-Trait-Anger-Expression-Inventory, and the Experiences in Close Relationships were not developed to assess personality functioning, but different cognitive or behavioral aspects. Stern et al. [22] used the Inventory of Personality Organization (IPO) [19] as criterion for concurrent validity testing of the STIPO scales“ identity”, “primitive defenses”, and “reality testing”. They found significant correlations between .45 and .57, which reflects the closeness of the two instruments: Both, the IPO questionnaire and the STIPO, are based on Kernberg’s concepts.

Another remarkable finding is that almost all STIPO dimensions showed their highest correlations with the ADP-IV “antisocial PD” scale. Looking at Kernberg’s concept of antisocial behavior in relation to personality organization, it is clearly regarded as a manifestation of a very low level of personality organization [5,6]. Since antisocial behavior is the key feature of antisocial personality disorder, it is not surprising that the “antisocial PD” score correlates highly with the STIPO dimensions. Interestingly, the STIPO dimension “moral values” shows the lowest correlations with all other questionnaire scales but the ADP-IV “antisocial PD” scale. This may indicate, that the converse argument does not hold. Low level of personality organization does not necessarily go along with antisocial behavior, while antisocial behavior is linked to low level of personality organization.

The concept of identity underlying the STIPO is also the basis of the BPI, thus, it has to be expected that the correlations of the STIPO “identity” domain with the BPI scales are high. The fact that the correlation with the BPI scales “primitive defenses” and “fear of closeness” were even slightly higher than the one with the BPI scale “identity diffusion” again reveals the fact that the different dimensions of Kernberg´s concept are not independent from each other, but rather based in a shared basic pathology. The same is true for “primitive defenses” that are closely related to identity diffusion in Kernberg’s concept.

The STIPO “object relations” scale shows comparably low correlations with the predicted questionnaire scales. This may have two reasons: Kernberg’s concept of object relations (and the corresponding STIPO scale) does not only contain cognitive and behavioral aspects, but also emphasizes affective components and internal working models of relationships. These are not (or much less) incorporated into the FSKN and ECR scales. The other reason for the lower correlations can probably be found in the fact that an impairment in this domain does not exclusively occur in patients with low personality organization, but also in patients with moderate impairment (neurotic level). Some features of the STIPO object relations domain are regarded as specific for low personality functioning (e.g., incapacity to be alone), while others are not (e.g., not having an intimate relationship for years).

The two STIPO subdomains of aggression, “self-directed” and “other-directed aggression”, correlate higher with the BPI and a few other scales than with the predicted FKBS and STAXI scales. This result can be explained by the different degrees or forms of aggression that are addressed: the STIPO asks for severe and partly physical aggression against the self and others, while the two questionnaires primarily focus less severe inner feelings of aggression or less severe verbal expression of anger towards others.

In addition to high correlations with the BPI scales and the ADP-IV “antisocial PD” scale, the STIPO domain “coping/rigidity” showed the predicted high correlation with the SPQ “negative coping” scale, whereas the predicted correlation with the FSKN “problem solving” scale was very low. A closer look at the FSKN scale reveals that it focuses a person’s self-image, while the STIPO are directed more towards behavioral aspects, how a person really copes with specific strains.

The low correlation of the STIPO “reality testing” domain with the corresponding BPI scale was somewhat surprising, since both instruments root in the same theory. However, a closer inspection of the BPI items reveals that the questionnaire solely addresses hallucinatory symptoms and thought disorder, while the STIPO aims at a broader spectrum of problems including paranoid thinking, dissociation, and depersonalization.

To summarize, the results demonstrated the convergent validity of the STIPO by means of a priori hypothesized correlations between the STIPO domains and related questionnaire scales. The correlations were not the highest of each STIPO domain with all of the questionnaire scales, which can be attributed to the different concepts underlying the instruments. As mentioned above, the highest correlations were found with the conceptually most closely related measures, the BPI and the ADP-IV “antisocial PD” scale.

The evaluation of discriminant validity is of particular importance here, since DSM-5 [15,16] has adapted the concept of personality functioning for the assessment of personality disorders. If the STIPO should be acceptable as an instrument for the determination of personality functioning in the sense of DSM-5 it must be able to differentiate between patients with and without personality disorder and between different degrees of severity among patients with personality disorders. Our results demonstrate that the STIPO can well distinguish between patients with and without personality disorder (between group effect size d=1.62) as well as between cluster B and cluster C personality disorder (d=1.26).

Taken together, the STIPO can be regarded as a reliable and valid instrument for the assessment of personality functioning in clinical and research settings. It might help to validate the DSM-5 Levels of Personality Functioning Scale [16] and other instruments - particularly questionnaires - that aim at the assessment of these dimensions. In a clinical setting, the duration of a STIPO interview (90 to 180 minutes) might be seen as a disadvantage. Therefore, short versions of the interview are presently developed. For research purposes, the STIPO appears to have two main advantages compared to other instruments: The structured interview type yields better interrater reliability and no audio or video recordings are needed for the ratings, which are done by the interviewer during the interview. In contrast, less structured interviews like SPC or OPD-2 require expert ratings of the recordings for the achievement of sufficient reliability of the ratings.

A highly interesting research question for the future will be, whether the DSM-5 restriction to two domains of personality functioning (self and interpersonal) is justified. Recently, it was argued, that additional dimensions of personality functioning, like e.g. aggression/ impulse control are needed [51]. On the one hand, the additional dimensions of the STIPO or related instruments provide important clinical information for indication and treatment planning. On the other hand, it remains to be investigated, whether the different dimensions change simultaneously during treatment or consecutively. It appears likely that, e.g., the sense of self and others changes before the quality of interpersonal relationships improves. It is to be expected that the DSM-5 focus on personality functioning will trigger research in this area that can benefit from reliable and valid instruments like the STIPO.

## Conclusion

The STIPO represents a reliable and valid instrument for the assessment of personality functioning that can be employed for clinical and research purposes. It might be particularly suited for research in the realm of the levels of personality functioning as defined by the upcoming DSM-5.

## Abbreviations

ADP-IV: Assessment of DSM-IV Personality Disorders Questionnaire; BoBi: Bochumer Bindungsfragebogen (German version of the ECR); BPI: Borderline Personality Inventory; DES: Dissociative Experiences Scale; DMI: Defense Mechanism Inventory; DSM-IV: Diagnostic and Statistical Manual of Mental Disorders – Fourth Edition; DSM-5: Diagnostic and Statistical Manual of Mental Disorders – Fifth Edition; ECR: Experiences in Close Relationships; FDS-20: Fragebogen zu dissoziativen Symptomen (modified German version of the DES); FKBS: Fragebogen zu Konfliktbewältigungsstrategien (Coping with Conflict Questionnaire); FSKN: Frankfurter Selbstkonzeptskalen (Frankfurt Self-Concept Scales); ICC: Intraclass Correlation; ICD-11: International Classification of Mental and Behavioral Disorders Tenth Revision; IPO: Inventory of Personality Organization; LPFS: Levels of Personality Functioning Scale; OPD: Operationalized Psychodynamic Diagnosis; OPD-2: Operationalized Psychodynamic Diagnosis Second Edition; PD: Personality Disorder; SCID-I: Structured Clinical Interview for DSM-IV Axis I; SCID-II: Structured Clinical Interview for DSM-IV Axis II; SPC: Scales of Psychological Capacities; SPQ: Stress-Process Questionnaire; STAXI: State-Trait-Anger-Expression-Inventory; STIPO: Structured Interview of Personality organization; SVF-120: Stressverarbeitungsfragebogen (German version of the SPQ); TFP: Transference-Focused Psychotherapy.

## Competing interests

The authors declare that they have no competing interests.

## Authors’ contributions

SD developed the study design, carried out the statistical analyses, and drafted the manuscript, ML, DM, BB, and MF recruited the patients, have been responsible for data acquisition, GS, MB, GH, and SD contributed substantially to data analyses and interpretation of data. All authors contributed substantially to a number of revisions of the manuscript and gave final approval for its submission. All authors read and approved the final manuscript.

## Pre-publication history

The pre-publication history for this paper can be accessed here:

http://www.biomedcentral.com/1471-244X/13/210/prepub
